# Trust in Medicine as a Factor Conditioning Behaviors Recommended by Healthcare Experts during the COVID-19 Pandemic in Poland

**DOI:** 10.3390/ijerph19010605

**Published:** 2022-01-05

**Authors:** Marta Makowska, Rafał Boguszewski, Monika Podkowińska

**Affiliations:** Department of Sociology, Institute of Sociological Sciences and Pedagogy, Warsaw University of Life Sciences, 02-787 Warsaw, Poland; rafal_boguszewski@sggw.edu.pl (R.B.); monika_podkowinska@sggw.edu.pl (M.P.)

**Keywords:** trust, COVID-19, behavior, healthcare, pharmaceutical industry, Poland

## Abstract

Objective: Due to the COVID-19 pandemic, public health experts have faced the challenge of convincing people to change their everyday habits. This study aims to evaluate the impact of trust in medicine on Polish citizens’ adherence to recommended behaviors. Methods: An online survey was conducted on a quota sample of adult Poles (*n* = 1072) during the second wave of COVID-19. Results: The trust-in-medicine index was created from statements relating to trust in healthcare professionals, vaccines, and medicines. This index showed that 27.1% of respondents expressed low trust, 36.7% expressed moderate trust, and 36.3% expressed high trust. The recommended behavior index was created from nine statements. This index showed that 15.8% of respondents had low adherence, 38.2% had moderate adherence, and 46.0% had high adherence to the healthcare experts’ recommendations. One-way analysis of variance showed that people with a high trust had significantly higher scores on the recommended behavior index when compared to people with a moderate or low trust. Conclusions: This study suggests that those responsible for health policy should put more effort into building trust not only in health professionals, but also in pharmaceutical companies. We also determined the socio-demographic features of people to whom such actions of trust building should be directed.

## 1. Introduction

December 2019, when the severe acute respiratory coronavirus 2 (SARS-CoV-2) was first detected in China, has already become a symbolic month, marking the beginning of immense, rapid changes all around the world. Many people have begun to experience anxiety [1,2,3,4], which is the natural correlate of all threats [5]. Lockdowns have been ordered in many countries, and new recommendations for pro-health behaviors have been introduced. These recommendations were supported by the voices of healthcare experts in an attempt to persuade the public to comply [6]. Social psychology has demonstrated that authority can have an enormous impact on the behavior of the average person [7,8]. Likewise, in the field of medicine, the power of authority is extremely visible [7]. Medical knowledge is constantly being updated. This knowledge is difficult for the average person to comprehend; therefore, people educated in this field should be trusted by the public. Heinzel and Liese wrote that authorities during the coronavirus disease 2019 (COVID-19) crisis have become a source of a heuristic [9], if we trust them, then we do not have to wonder what is right—we simply follow their advice. However, the erosion of scientific authority has also been occurring for several years [10,11]. The dissemination of conspiratorial theories has challenged scientific knowledge. “Conspiracy theories” can be defined as “modes of thinking, templates imposed upon the world to give the appearance of order to events” [12] (p. 2). There is no shortage of such theories concerning the medical world, especially the pharmaceutical industry. Some conspiracy theories maintain that vaccinations cause autism or that big pharma withholds cures to keep people on more expensive, less effective treatments [13].

In our opinion, trust in medicine consists not only of trust in healthcare professionals but also in the products of pharmaceutical companies (such as vaccines or medicines). The COVID-19 pandemic has shown that trust in the pharmaceutical industry and its products is eminently important. At the time of the writing of this article, there is a struggle in many countries to convince as many citizens as possible to get vaccinated; to be convinced, one must believe that the products of pharmaceutical companies are safe and effective. “The anti-vaccination movement, alternative medicine or conspiracy theories related to big pharma are current and often life-threatening dangers” [14] (p. 5). The success of preventative measures against COVID-19 may depend upon the extent to which a given society trusts and adheres to the recommendations of healthcare experts.

Trust is a sociological construct around which many definitions and theories have been developed [15,16,17,18]. When one considers trust in medicine in the present world of uncertainty, the following definition is applicable: “Trust means having confidence in ‘abstract systems’—for example, we have to have confidence in agencies for food regulations, the purification of the water […] Trust and risk are closely bound up with one another. We need to have confidence in such authorities if we are to confront the risks which surround us” [16] (pp. 123–124). Rhodes and Stain wrote that it is imperative that we put trust in individuals and institutions related to health, but healthcare institutions are not designed to promote trust [19].

Over the years, pharmaceutical companies have not acted in ways that would help to gain greater social trust [20,21]. The latest Edelman Trust Barometer indicated that the pandemic has increased distrust in community institutions and leaders around the world. People believe that governments, businesses, and journalists were deliberately reporting false information. The healthcare industry also experienced a decline of 2% on this barometer since 2019 [22].

In Poland, problems with trust are evident. Polish culture has even been called a “culture of distrust” [18]. Research has shown low rates of social trust in Poland over a span of many years [23]. In 2021, YouGov conducted an international survey that showed that Poland is the only country in which healthcare professionals are not the most trusted people; family and friends, are the most trusted in Poland [24].

Our study was conducted at a time when Poland faced the second wave of the coronavirus. At the peak of this wave, there were 37,596 cases a day [25]. The lockdown implemented introduced the following rules: distance learning for schoolchildren, the closure of cultural institutions, restrictions on the operation of hotels, the suspension of sanatoriums, limiting the functioning of shopping malls, significant restrictions on the number of people allowed in shops, churches, and public transport, restrictions on public gatherings, bans on organizing meetings and events, and the suspension of activities at swimming pools, aquaparks, and gyms. In addition, there was an order to wear masks in public places [26]. Poles’ compliance with government restrictions and the recommendations of healthcare experts seemed to be crucial in the fight against the virus, and arguments were presented in media to encourage their compliance.

The main goal of this article is to evaluate the impact of trust in medicine on adherence to recommended behaviors in Poland. When conducting the research, we wanted to answer three main research questions: (1) How many Polish people trust in medicine during the second wave of the pandemic? (2) What socio-demographic factors explain trust in medicine? (3) Does trust in medicine have an impact on adherence to recommended behaviors during the pandemic?

## 2. Materials and Methods

An online survey was conducted between 24 and 27 November 2020. It is worth noting that the scope of the study was wider than that to which this article is devoted. It concerned the pandemic-related attitudes and behavior of Poles. The survey had 11 main questions and 19 socio-demographic questions. The questionnaire was created in line with the procedures outlined by Malhotra [27]. Content validity was ensured via pilot testing and consultations with experts. “Assessing content validity does not depend on empirical support, just logical justification” [28] (p. 45). The results of the pilot testing and the experts’ feedback were incorporated while designing the final version of the questionnaire. The aim was not to create a standard tool for testing attitudes or to adapt existing tools. The multifaceted nature of the research makes it more like an omnibus study than a standardized study of specific attitudes. The individual scales built for the purpose of this study based on the indicators included in the survey were subject to statistical validation. In addition, Poland was the only country in which the research was carried out; thus, no validation issues relating to adaptation to the culture or translation arose. The questionnaire and database are publicly available on the figshare website: https://figshare.com/articles/dataset/Poland_Covid-19_3rd/14398835 (accessed on 4 January 2022). Additionally, the questionnaire is reprinted in Supplementary Material A.

Based on t-tests carried out in G * Power 3.1 [29], assuming an alpha of 0.05, a desired power of 0.80, a (small) effect size of d = 0.2, an allocation ratio of 0.25, and that 20 million adult Poles have access to the Internet, we should have conducted the research using 968 respondents. Hence, we aimed to gather data from more than 1000 respondents. Ultimately, 1072 respondents took part in the study. The quota sample was consistent with the distribution of the following characteristics in the population of adult Poles: gender (two groups), age (two groups), province (sixteen groups), population size of place of residence (four groups), and education (two groups: higher and other). Supplementary Material B provides an exact breakdown of the socio-demographic characteristics of the respondents.

Ethics committee consent was not obtained, as Polish regulations do not require this for online sociological surveys carried out anonymously on samples of adults with their consent. In addition, at the time of the study, there was no ethics committee at our university that could consider the research. However, because we used the SW Research Agencja Badań Rynku i Opinii Internet Research Panel (one of the largest in Poland), our research was done in accordance with their policies, which meet the highest research standards (see https://swresearch.pl/o-firmie/jakosc; accessed on 4 January 2022). The participants received points for participating in the study, which they could then exchange for awards in the SW Research rewards pool.

IBM SPSS Statistics (SPSS Inc., Chicago, IL, USA; version 27) was used to perform the various statistical analyses. The simplest of these were frequency distributions. However, the main conclusions in this study are drawn from the construction of two indices—the trust index and the recommended behaviors index. Cronbach’s alpha coefficients were computed for both. Multiple linear regression was then performed to check which socio-demographic characteristics of the respondents favored an increase in the trust index value (continuous variable). To investigate whether there was a relationship between the distinguished groups with different trust in medicine and the recommended behaviors index (continuous variable), a one-way analysis of variance (ANOVA) was performed. Additionally, between the two indices (continuous variables), we conducted a Pearson’s correlation to investigate the strength of the relationship between the two dimensions analyzed.

## 3. Results

From our questionnaire, based on an arbitrary assessment, we selected statements related to trust in medicine. Initially, there were six statements selected (see Supplementary Material A—Q4: 15–20). The arbitrary selection of statements was verified by reliability analysis. Various combinations of statements led to the rejection of two statements, allowing the Cronbach’s alpha coefficient to rise from 0.63 to 0.74.

The frequency distributions of the four statements that remained are presented in Table 1. More than a quarter of the respondents (26.7%) agreed that “Healthcare professionals should not be completely trusted.” The opposite opinion was expressed by 40.0% of respondents. Almost every fourth respondent (23.6%) agreed with the statement that “In the current situation, healthcare professionals are not trying as much as they should”, while almost half of the respondents (47.2%) disagreed with this statement. With the statement “I think the anti-vaccine movement is right”, 22.2% of respondents agreed, while 45.4% of respondents expressed the opposite opinion. With the last statement, “Natural treatments (e.g., herbs and a good diet) are more effective than medications”, 20.2% of respondents agreed, and 43.4% disagreed. It is worth noting that, for all statements, there was a very large group of people (this number was always close to 30.0%) who chose the “hard to say” option.

From these four statements, we created a trust index. For each statement, for the answer “definitely agree”, the respondent received one point, for “probably agree”, two points, for “hard to say”, three points, for “probably disagree”, four points, and for “definitely disagree”, five points. The trust index calculated the mean of all four statements for each respondent. A higher mean meant a higher trust in medicine. Respondents who obtained a mean from 1 to 2.75 were included in the group of people with low trust, from 2.76 to 3.5 were in the group with moderate trust, and those from 3.51 to 5 were in the group with high trust. As much as 27.1% had a low trust in medicine, 36.7% had a moderate trust, and 36.3% had a high trust.

To identify the socio-demographic characteristics that significantly promote the growth of trust in medicine, a linear regression analysis was carried out. The multiple regression model taking into account the introduced predictors indicates that the probability of achieving higher index values (greater trust) occurs in men, residents of places with larger population sizes, respondents with fewer children in the household, people dissatisfied with their health, people with a better financial situation, people less likely to practice religion, and respondents who have been in contact with COVID-19 (they were ill or someone from their family or friends was).

These features were found to be statistically significant, while the whole model is well suited to the data and allows for better prediction than achieved with mean comparisons: F (9, 1062) = 13.60; *p* < 0.001; R^2^ = 0.103. Detailed results of the analysis are presented in Table 2.

Based on the data from our study, we also built a recommended behaviors index. Statements that referred to recommended behaviors were arbitrarily selected from our survey. Initially, there were 11 statements selected (see Supplementary Material A—Q5 1–5; 7–10; 14); finally, based on the reliability analysis, in which Cronbach’s alpha coefficient was reasonable (0.80), nine of them remained. The statements and their frequency distributions are presented in Table 3. A recommended behavior to which most respondents (80.3%) adhered was wearing a mask in every situation where it was recommended by the government. More than half of Poles (66.3%) were trying to take care of their immunity better by engaging in appropriate healthy behavior. A similar percentage of respondents (63.3%) agreed with the statement, “If I developed coronavirus symptoms, I would immediately contact a physician.” Adherence to the restrictions imposed by the government was declared by 60.6% of respondents, and 56.9% said that, despite the defrosting of the economy, they still tried to limit unnecessary activities and contacts. As many as 44.9% of Poles did not regularly meet with friends and family outside their household, and 39.8% gave up offering a hand to greet anyone except members of their household (shaking hands is a traditional welcoming gesture in Poland). Almost the same number (39.2%) acquired appropriate food supplies to allow themselves to stay at home. The rarest recommended behavior declared by Poles was the declaration to vaccinate—this was stated by only 30.1% of respondents.

To create a recommended behaviors index, the 5-point Likert scale was inverted in all statements except for “I meet my friends and family outside my household quite regularly”. This means that, in the remaining eight statements, one point was awarded for the answer “I definitely disagree” and five points for the answer “I definitely agree”. By this measure, a higher value of the recommended behavior index meant greater adherence to recommendations. The recommended behavior index was used to calculate the mean from all nine statements for each respondent. Based on the calculated means, we divided the respondents into three groups. The first group consisted of people with low adherence to recommended behaviors. This group constituted 15.8% of respondents and included those whose mean was an index range from 1 to 2.75. The second group consisted of people with moderate adherence to recommended behaviors. Their mean in the index ranged from 2.76 to 3.5. This group constituted 38.2% of the respondents. The third group consisted of people with high adherence to recommended behaviors. Their mean ranged from 3.51 to 5. Most of the respondents (46.0%) were in this last group.

One-way ANOVA showed a significant main effect of trust in medicine on the recommended behavior index. A post hoc Games–Howell test showed that people with high trust scored significantly higher in the recommended behavior index than did people with moderate or low trust. People with high trust earned significantly higher scores in the recommended behavior index than did people with low trust. This means that the higher the trust in medicine, the greater the compliance with pro-health recommendations (see Table 4). In addition, the results of the Pearson’s correlation indicated that there was a significant medium positive association between the trust index and recommended behavior index (r = 0.337, *p* < 0.001). Thus, if someone got higher scores on the trust index (continuous variable), he or she would also get higher scores on the recommended behavior index (continuous variable).

## 4. Discussion

Our study shows that during the second wave of the COVID-19 pandemic, 36.7% of Poles had a high trust in medicine and 36.3% had a moderate trust. The group with a low trust was the smallest group, but still considerable (27.1%). The limited trust of Poles in medicine during the pandemic may be explained by the fact that Poles did not show much confidence in doctors before the current public health crisis [30,31,32]. In addition, as mentioned in the introduction, Poles are generally characterized by a low social trust [18,23].

The public health emergency caused by the COVID-19 pandemic highlighted the problems with which the Polish health service have been struggling with for years, including staff deficits [33,34] and underfunding [33]. During the pandemic, in cases unrelated to COVID-19, 74.9% of Poles had difficulty accessing a doctor [35]. This certainly did not have a positive effect on trust.

In our study, women, people from places of residence with smaller population sizes, those with worse financial situations, and those who more frequently participated in religious practices had a lower trust in medicine. All of these groups are generally characterized by lower social trust in Poland [23]. It is also worth noting that there are studies on trust in doctors from other countries showing similar relationships. Some show, as did our research, that females are less trusting in doctors [36,37], as are rural residents [36], people with lower incomes [37], and people who more frequently participate in religious practices [38].

Our research also shows that people who assess their health condition as good have a lower trust in medicine. The same situation is true of respondents who did not have contact with the coronavirus (were not infected themselves/they did not have someone from their family or close friends infected). It can therefore be assumed that such people did not have personal contact with the health service during the pandemic and, thus, their assessments are not based on experience, but only selective reports, which may increase distrust. Research confirms that a recent experience of hospitalization or a medical visit has a positive effect on trust in a doctor [37]. This thread, however, requires deeper and further research into the Polish sociocultural reality.

The surveyed Poles complied with the recommended behaviors to a varying degree; although, importantly, the low-adherence group was the smallest one (15.8%). The group with moderate adherence constituted 38.2% of the respondents. The largest group (46.0%) was that with high adherence. Most respondents (80.3%) declared compliance with the recommendation to wear a mask in every situation where it was recommended by the government. This fact is not surprising, as it was a legal obligation during the study [39].

It is worth emphasizing that the desire to get vaccinated was the least frequently declared recommended behavior by Poles; only 30.1% of respondents stated this. There were no vaccinations available at the time of the study, so the vaccination situation was only hypothetical. At the time of the writing of this article, there is a struggle in many countries to persuade as many citizens as possible to get vaccinated. For this, the belief that the products of pharmaceutical companies are safe and effective and high trust in health authorities are needed. Currently, 54% of Polish society is fully vaccinated [40]. This percentage is larger than the number people who declared their willingness to be vaccinated in our study (it is worth noting that the data on the Ministry’s website concerned all Poles, while we studied the 18+ population.). This may mean that, since vaccines became available, extensive action by the authorities to convince the population of their safety has had a positive effect, but only on a part of society.

It is worth considering where the reluctance to comply with the recommendations may come from. People deal with a pandemic and the threat it has created in different ways. One of the many ways to deal with the stress created by pandemic can be denial—“a refusal to accept external reality because it is too threatening, which can reduce anxiety” [41] (p. 43). This strategy may lead to the recognition of any precautions as unnecessary.

Our research has shown that trust in medicine has an impact on adherence to recommended behaviors. People with a high trust in medicine achieved significantly (*p* < 0.01) higher values on the recommended behavior index (M = 3.65) than did people with a moderate (M = 3.31) or low (M = 3.07) trust. The results of the Pearson’s correlation also indicated a medium positive association between the trust index and the recommended behavior index (r = 0.337, *p* < 0.001). Adapting to the recommendations of authorities can directly translate into reducing the rate of viral spread and into health outcomes. The meta-analysis by Birkhäuer et al. [42], taking into account 47 studies, found the existence of a correlation (small-to-moderate) between trust in healthcare and health outcomes.

A lack of trust (in doctors, entities involved in vaccine development, or healthcare) has already been linked in some studies with higher levels of COVID-19 vaccine hesitancy [43,44], and trust in health authorities and experts with a higher likelihood of vaccination [45,46]. An Italian study also showed that pregnant women were more likely to take the influenza and Tdap (tetanus, diphtheria, pertussis) vaccines when they were recommended by a healthcare provider, and the main reason not to get vaccinated was the lack of such a recommendation. Interestingly, the conclusion of this latter study was that the COVID-19 pandemic experience had a positive impact on raising awareness of the role of vaccination in preventing diseases during pregnancy [47].

Earlier analyses conducted during the COVID-19 pandemic showed that people who believe in conspiracy theories are less compliant with health recommendations [48,49,50]. Research conducted in Poland at the same time as the current study showed that 31% of Poles believe that “The coronavirus epidemic for the health of Poles is an exaggerated threat”, and 3% think that it is a fictional threat. As many as 28% of Poles agreed with the statement “The coronavirus pandemic was artificially triggered to reduce the human population on Earth”, and 45% agreed that “The pharmaceutical lobbies, politicians and the media around the world are deliberately exaggerating the dangers of the coronavirus” [51]. Such pandemic-related conspiracy narratives are typical and can be interpreted as an expression of suspicion towards scientific knowledge. They are often accompanied by actions that are critical to expert messages [52], with skeptics indicating that they do not want to be “puppets” or to “lose their freedom”. One recommended way to combat misinformation is to present scientific evidence in a simple way to avoid misinterpretation and misunderstandings [53].

Research has also shown that confidence in the government [54], institutional trust [55], and healthcare system trust [56,57,58] were associated with a higher subordination of the recommended behaviors during the COVID-19 pandemic. We also found studies that, like ours, show that trust in medicine (understood as trust in doctors) increases compliance with preventative behavior [59]. However, in our study—compared to the one cited above—trust was defined more broadly. We created a complex indicator and defined trust as trust in healthcare professionals, vaccinations, and medications. Similar studies have not been conducted in Poland before, and the results may depend on the cultural and social context. Polish society seems extremely interesting in this respect, considering the pre-pandemic problem Poles have with trust [18,23].

The unique time in which this research was carried out—during the second wave of the pandemic in Poland—can be treated both as a strength (uniqueness of the situation) and as a limitation of our research (it was a time snapshot). During the study, the number of daily reported cases of infections and deaths exceeded the previously reported maximums. After the initial, very strict compliance with governmental recommendations [60], Polish society got used to the situation and was no longer willing to follow restrictions thoughtlessly, as in the case of the first wave.

Another limitation of our study was that it was conducted on an online quota sample. This means that our sample was not fully representative. Still, about 30% of Poles do not use the Internet regularly [61], and this could have influenced the results we obtained. Another limitation was that our research project was broad and focused on many issues related to the pandemic, and it was not strictly focused on trust. Therefore, we could not create other indicators, for example, relating to trust in science, government, or pharmaceutical companies. Certainly, as these threads are extremely important and interesting, they should be raised in subsequent studies on the pandemic in Poland. Our study is also a social study based on respondents’ declarations, and it should be remembered that sometimes people have difficulty admitting undesirable behavior. Hence, these results will not be as accurate as, for example, those obtained based on observation. Finally, many different factors in addition to trust that we did not take into account in our study can affect a greater propensity toward recommended behaviors—for example, personality factors.

## 5. Conclusions

Our study confirms that trust in medicine can have significant importance in fighting the COVID-19 pandemic. People who scored higher on the trust index also had higher adherence to the recommended behaviors. It can therefore be suggested to those responsible for health policy that they focus on building trust—not only in health professionals, but also in pharmaceutical companies. In our study, we identified who such actions should be primarily targeted at in Poland: women, people from smaller population centers, those with a worse financial situation, frequent religious practitioners, those less satisfied with their health condition, and those who were not infected with coronavirus themselves and whose family or friends were not infected. Based on the results of our research, it is necessary to agree with the statement of Gopichandran, Subramaniam, and Kalsingh that, during the COVID-19 pandemic, “building trust and confidence therefore becomes an ethical imperative.” [57].

## Figures and Tables

**Table 1:** Distributions of responses to statements used to create the trust index (*n* = 1072).

	Definitely Agree % (*n*)	Probably Agree % (*n*)	Hard to Say % (*n*)	Probably Disagree % (*n*)	Definitely Disagree % (*n*)
Healthcare professionals should not be completely trusted	8.1 (87)	18.6 (199)	33.3 (357)	25.0 (268)	15.0 (161)
In the current situation, healthcare professionals are not trying as much as they should	10.4 (112)	13.2 (142)	29.1 (312)	24.9 (267)	22.3 (239)
I think the anti-vaccine movement is right	9.0 (97)	13.2 (141)	32.5 (348)	18.4 (197)	27.0 (289)
Natural treatments (e.g., herbs, and a good diet) are more effective than medications	6.2 (66)	14.0 (150)	36.5 (391)	25.3 (271)	18.1 (194)

**Table 2:** The results of the linear regression analysis predicting the trust index score (continuous variable) from socio-demographic characteristics (*n* = 1072).

Predictor	B	SE B	β	*t*	*p*
Gender (female–male)	0.104	0.052	0.058	1.987	**<0.05**
Age group (increasing)	0.016	0.022	0.024	0.737	0.461
Population size of place of residence (increasing)	0.084	0.019	0.136	4.507	**<0.001**
Education (increasing)	0.040	0.021	0.055	1.855	0.064
Number of children (under 18 years of age) living in household (increasing)	−0.070	0.035	−0.059	−1.983	**<0.05**
Self-assessment of health (bad–good)	−0.082	0.040	−0.072	−2.046	**<0.05**
Financial situation of household (bad–good)	0.092	0.039	0.078	2.349	**<0.05**
Participation in religious practices when not socially isolating (increasing)	−0.118	0.018	−0.200	−6.714	**<0.001**
Respondent or someone from family or close friends was infected (no–yes)	0.221	0.062	0.104	3.547	**<0.001**
Constant	2.357	0.171		13.750	**<0.001**

B—unstandardized regression coefficient, SE—standard error, β—standardized regression coefficient, *t*—*t*-test value, *p*—significance level. Bold font indicates statistical significance.

**Table 3:** Distribution of responses to statements used to create the recommended behaviors index (*n* = 1072).

	Definitely Disagree % (*n*)	Probably Disagree % (*n*)	Hard to Say % (*n*)	Probably Agree % (*n*)	Definitely Agree % (*n*)
I strictly adhere to the restrictions imposed by the government in the fight against the pandemic	5.6 (60)	11.7 (125)	22.1 (237)	44.0 (472)	16.6 (178)
I have acquired appropriate food supplies to allow myself to stay at home for a long period of time	12.5 (134)	31.0 (332)	17.4 (186)	30.9 (331)	8.3 (89)
I wear a mask in every situation where it is recommended by the government	3.2 (34)	6.5 (70)	10.0 (107)	34.6 (371)	45.7 (490)
In the current situation, I would not offer my hand to greet anyone except members of my household	14.3 (153)	23.0 (247)	22.9 (245)	26.4 (283)	13.4 (144)
If I developed coronavirus symptoms, I would immediately contact a physician	4.6 (49)	10.4 (112)	21.5 (230)	38.1 (408)	25.5 (273)
I would get vaccinated if a coronavirus vaccine was already available	25 (268)	15.3 (164)	29.7 (318)	17.1 (183)	13.0 (139)
I am now trying to take care of my immunity better by engaging in appropriate healthy behavior	2.1 (22)	112 (10.4)	21.2 (227)	506 (47.2)	19.1 (205)
Despite the lifting of restrictions to defrost the economy, I am still trying to limit unnecessary contacts and activities	7.1 (76)	16.9 (181)	19.1 (205)	40.8 (437)	16.1 (173)
I meet my friends and family outside my household quite regularly	12.6 (135)	32.3 (346)	18.1 (194)	27.1 (291)	9.9 (106)

**Table 4:** Results of one-way ANOVA on the recommended behavior index (continuous variable) for groups differing in trust in medicine.

Independent Variable	*n*	M	SD	F	*p*	Post Hoc Comparisons (Games–Howell Test *)		
							Mean Difference	*p*
Low trust	290	3.07	0.82	61.9 (2, 1069)	<0.01	Moderate trust	−0.24	**<0.01**
						High trust	−0.58	**<0.01**
Moderate trust	393	3.31	0.60			Low trust	0.24	**<0.01**
						High trust	−0.33	**<0.01**
High trust	389	3.65	0.63			Low trust	0.58	**<0.01**
						Moderate trust	0.33	**<0.01**

* The Games–Howell test was used due to the nonfulfillment of the condition of equal variance. Bold font indicates statistical significance.

## Data Availability

Data available in a publicly accessible repository. The data presented in this study are openly available in figshare at https://figshare.com/articles/dataset/Poland_Covid-19_3rd/14398835 (accessed on 4 January 2022).

## Supplementary Materials

The following supporting information can be downloaded at: https://www.mdpi.com/article/10.3390/ijerph19010605/s1, Supplementary Material A: Questionnaire—3rd wave; Supplementary Material B: Final cluster centers resulting from a cluster analysis using the k-means method.
