# Supplementary material for: Trust in Medicine as a Factor Conditioning Behaviors Recommended by Healthcare Experts during the COVID-19 Pandemic in Poland
Source: Int J Environ Res Public Health. 2022 Jan 5;19(1):605. doi: 10.3390/ijerph19010605 (PMC8744838; doi:10.3390/ijerph19010605)
Supplement: Supplementary file 1 [file ijerph-19-00605-s001.zip › Supplementary Material A.pdf]

Questionnaire – 3rd wave

Good day,

We encourage you to participate in our scientific study of the current problems in Poland and the rest of the world. The survey is anonymous, and its results, in the form of aggregated summaries, will be used solely for scientific purposes. The survey will take about 15 minutes to complete.

Your opinions are extremely important to us, so please be serious in your approach to the topic and give honest answers.

Thank you in advance for participating in the survey.

Monika Podkowińska, PhD hab., Marta Makowska, PhD, Rafał Boguszewski, PhD
Staff of the Institute of Sociological Sciences and Pedagogy at SGGW

Q1. Together with its positive effects, many negative phenomena and threats have accompanied the development of civilization. Which of the following phenomena would you consider to be the most dangerous? **Please select a maximum of 3 answers (items 1 to 10 were listed in a rotated order for each presentation of the questionnaire)**

1) Environmental pollution
2) The use of chemical compounds and genetic modifications in food products
3) Diseases associated with civilization (e.g., cancer, hypertension, obesity)
4) Outbreaks and pandemics of previously unknown viruses
5) Climate change
6) Poverty
7) Unemployment
8) Exhaustion of non-renewable energy sources
9) The increasing world population
10) Terrorism
11) Other phenomena/threats (please specify) ………………………...
12) Hard to say

Q2. We are currently in an unusual situation: the quarantining of our entire society due to the coronavirus pandemic. Please indicate the degree to which each statement applies to you. (The following items were listed in a rotated order for each presentation of the questionnaire).

	1 Definitely yes	2 Probably yes	3 Hard to say	4 Probably not	5 Definitely not
1) I feel fear for my health	1	2	3	4	5
2) I feel fear for the health and life of my loved ones	1	2	3	4	5
3) I am afraid that I will be financially broken by the prolonged pandemic	1	2	3	4	5
4) I am afraid of losing my job because of the situation (*for this statement,*	1	2	3	4	5

participants could also select 6 - not applicable)					
5) The prolonged period of social isolation is negatively affecting my mental well-being	1	2	3	4	5
6) Faith in God is helping me get through the pandemic	1	2	3	4	5

Q3. How the restrictions introduced by the government (including leaving home among things) have affected your relationships with the following people:

	Definitely worsened	Worsened a bit	Remained unchanged	Improved a bit	Definitely improved	Not applicable
1) Partner; husband/wife						
2) Children						
3) Extended family						
4) Friends/acquaintances						
5) Neighbors						
6) Colleagues						

Q4. To what extent do you agree with the following statements? (The following items were listed in a rotated order for each presentation of the questionnaire).

	1 Definitely yes	2 Probably yes	3 Hard to say	4 Probably not	5 Definitely not
1. The restrictions introduced by the government in the fight against the pandemic are too strict	1	2	3	4	5
2. People who let their children go to school are acting irresponsibly in the present situation	1	2	3	4	5
3. The period of social restrictions imposed by the government in the fight against the virus should not be extended any further	1	2	3	4	5
4. I believe that, for the good of the economy, decisions should not be taken to close borders and many institutions	1	2	3	4	5
5. The coronavirus is part of the political and economic war between the US and China	1	2	3	4	5
6. The virus was deliberately released to reduce the problem of overpopulation in the world	1	2	3	4	5
7. The global economy will recover quickly after the pandemic has been fought	1	2	3	4	5

8. The media have unnecessarily spread panic in society by exaggerating the situation	1	2	3	4	5
9. The pandemic will strengthen solidarity in the country	1	2	3	4	5
10. Thanks to the pandemic, people will understand what is really important in life	1	2	3	4	5
11. God is using the current situation to try to speak to people who have turned their backs on him	1	2	3	4	5
12. I think that the defrosting of the economy and lifting of restrictions is happening too quickly	1	2	3	4	5
13. Coronavirus is nothing more than a worse type of flu	1	2	3	4	5
14. In order not to get infected with coronavirus, it is enough to wash your hands often and avoid large clusters of people	1	2	3	4	5
15. Healthcare professionals should not be completely trusted	1	2	3	4	5
16. I believe that Primary Healthcare is currently not functioning efficiently in Poland	1	2	3	4	5
17. I am concerned about having contact with healthcare professionals due to the coronavirus	1	2	3	4	5
18. In the current situation, healthcare professionals are not trying as much as they should	1	2	3	4	5
19. I think the anti-vaccine movement is right	1	2	3	4	5
20. Natural treatments (e.g., herbs, and a good diet) are more effective than medications	1	2	3	4	5
21. I believe that the coronavirus does not exist	1	2	3	4	5
22. Pharmaceutical companies are responsible for releasing the coronavirus	1	2	3	4	5

Q5. Please indicate the degree to which the following statements apply to you. (The following items were listed in a rotated order for each presentation of the questionnaire).

	1 Definitely yes	2 Probably yes	3 Hard to say	4 Probably not	5 Definitely not
1. I strictly adhere to the restrictions imposed by the government in the fight against the pandemic	1	2	3	4	5

2. I have acquired appropriate food supplies to allow myself to stay at home for a long period of time	1	2	3	4	5
3. I wear a mask in every situation where it is recommended by the government	1	2	3	4	5
4. In the current situation, I would not offer my hand to greet anyone except members of my household	1	2	3	4	5
5. I meet my friends and family outside my household quite regularly	1	2	3	4	5
6. I follow information about the pandemic daily, and monitor incidence statistics	1	2	3	4	5
7. If I developed coronavirus symptoms, I would immediately contact a physician	1	2	3	4	5
8. I would get vaccinated if a coronavirus vaccine was already available	1	2	3	4	5
9. I am now trying to take care of my immunity better by engaging in appropriate healthy behavior	1	2	3	4	5
10. I try to go for a walk regularly or engage in other outdoor activities	1	2	3	4	5
11. I try to get involved in social campaigns to support those who particularly need help in the current situation	1	2	3	4	5
12. I now devote more time than before to prayer and other religious practices	1	2	3	4	5
13. Currently, I am much less rigorous than before when adhering to the recommendations regarding leaving home and meeting other people	1	2	3	4	5
14. Despite the lifting of restrictions to defrost the economy, I am still trying to limit unnecessary contacts and activities	1	2	3	4	5
15. I know exactly what to do if I observe coronavirus symptoms in myself or members of my household	1	2	3	4	5

Q6. In connection with the Constitutional Tribunal’s judgment limiting the availability of abortion, some people, especially women, have protested on the streets to express their objections. Please indicate the extent of your agreement with the following statements.

	1 Definitely agree	2 Probably agree	3 Hard to say	4 Probably disagree	5 Definitely disagree
1. People who take part in the demonstrations are exposing society to an increase in coronavirus infections.	1	2	3	4	5
2. I think that demonstrations are necessary in order to make the government aware that many people do not agree with the Constitutional Tribunal’s judgment.	1	2	3	4	5

3. The Constitutional Tribunal’s judgment was right.	1	2	3	4	5
4. Polish abortion laws should be liberalized. Abortion should be available on demand.	1	2	3	4	5
5. It was not good timing for the Constitutional Tribunal to issue its judgment during the pandemic.	1	2	3	4	5
6. Poland’s abortion laws should be maintained in their current state. The so-called abortion compromise should not be changed.	1	2	3	4	5
7. Protesters should not use profanities.	1	2	3	4	5
8. Protesters should not destroy church property.	1	2	3	4	5
9. Protesters should not offend priests and bishops.	1	2	3	4	5

Questions 7 to 11 were about the social understanding and perceptions of home (excluded from publication on figshare on the request of one of the authors - MP)

Script for obtaining socio-demographic information:

M1. Gender
1. Woman
2. Man

M2. What year were your born? …………………………

M3. What is your highest level of education?
1. Primary
2. Gymnasium
3. Vocational
4. Secondary
5. Bachelor’s degree
6. Master’s degree
7. Doctorate
8. Single
9. Married
10. Divorced
11. Widow/widower

M5. How many adults (including you) live in your household? ….

M6. How many children (under 18 years of age) live in your household? …. **(do not ask if M5 = 1)**

M7. What is your current professional situation?
1. I work full-time as before
2. I work full-time remotely
3. I work as before, but for a shorter amount of time
4. I work remotely for a shorter amount of time
5. I am receiving care allowance

6. I am on holiday leave
7. I am on unpaid leave
8. I have been unemployed for more than 12 months (I lost my job before the pandemic)
9. I have been unemployed for less than 12 months (I have lost my job during the pandemic)
10. I am retired
11. I am a student
12. Other situation, please specify...

Ask M7A if M7 = 1,2,3,4

M7A. Are you employed in:

1. A public institution
2. A private company
3. A company with mixed-capital
4. Your own business

M8. In what type of area do you live?

1. Village
2. City of up to 19,999 residents
3. City 20,000 – 199,999 residents
4. City 200,000 – 499,999 residents
5. City with over 500,000 residents

M9. How do you assess your health?

1. Very good
2. Good
3. Moderate
4. Poor
5. Very poor

M10. How do you assess the financial situation of your household?

1. Very good
2. Good
3. Moderate
4. Poor
5. Very poor

M11. How do you think that the current situation in the country relating to the coronavirus pandemic will affect the financial situation of your household?

1. The situation will get a lot worse
2. The situation will get a bit worse
3. The situation will remain unchanged
4. The situation will improve a bit
5. The situation will improve a lot
6. Hard to say

M12. What is your religious denomination?

1. Catholicism
2. Another Christian denomination (Protestantism, Orthodoxy)
3. A non-Christian denomination (e.g., Judaism, Islam)
4. I do not profess any religion

M13. How often do you usually attend masses, services or other religious meetings? (when not socially isolating)

1. Several times a week
2. Once a week
3. 1-2 times a month
4. Several times a year
5. Once every few years
6. Not at all

M14. During the pandemic, have you sometimes participated in masses and other religious services held in a church?

1. Yes, regularly (at least once a week)
2. Yes, but not regularly (1-2 times a month)
3. Yes, but occasionally (less than once a month)
4. No.

M15. During the pandemic, have you ever participated in masses and other religious services transmitted via:

	1<br>Yes, regularly (at least once a week)	2<br>Yes, but not regularly (1-2 times a month)	3<br>Yes, but occasionally (less than once a month)	4<br>No
1. Television	1	2	3	4
2. Radio	1	2	3	4
3. The Internet	1	2	3	4

If M13 = 6 & M14 = 4 & M15 2 = 4 & M15 3 = 4 then do not ask M16 and M17

M16. Do you think that participating in a mass or other religious service using media such as the Internet, radio, and television…

	1.Yes, similar	2. No, less important/a worse experience	3. No, more important/ a better experience	4. Hard to say
Is as important as direct participation in a church	1	2	3	4
Gives the same religious experience as direct participation in a church	1	2	3	4

M17. Has your religious commitment changed during the coronavirus pandemic?

1. Yes, nowadays I spend more time in prayer, meditation and other religious practices
2. Yes, I spend less time now praying, meditating and other religious practices
3. No, I spend the same amount of time in prayer, meditation and other religious practices
4. Hard to say
5. Not applicable. I do not practice and have not practiced religion.

M18. Are/were you or any of your family or close friends:

	Yes	No	Don’t know

Infected with coronavirus			
Under quarantine			

M19. In general, are you satisfied with your life?

1. Definitely yes
2. Probably yes
3. Probably not
4. Definitely not
5. Hard to say

M20. How do you mainly obtain information on the spread of the coronavirus and the pandemic situation? Please indicate a maximum of two main sources.

1. Television (which station do you watch most often? – please select one): TVP (including TVP Info), TVN (including TVN24), Polsat (including Polsat News), TV Trwam, Other (please specify)
2. Radio
3. The press
4. Online news websites (which do you use most often? ………………– please give no more than two)
5. Social networking sites on the Internet
6. Friends and family
7. Other sources, please specify.................................
8. I do not seek out this information

M21. Which political grouping would currently have the best chance of having your support in Sejm elections? (1 items 1 to 5 were listed in a rotated order for each presentation of the questionnaire)

1. Law and Justice (Law and Justice, United Poland, Coalition)
2. Civic Coalition (Civic Platform, Modern, Polish Initiative, the Greens)
3. Democratic Left Alliance ("Left" – Democratic Left Alliance, Spring, Left Together)
4. Freedom and Independence Confederation (KORWiN, National Movement, Braun)
5. Polish People's Party ("PSL-Polish Coalition" – Polish People's Party, Kukiz'15)
6. Poland 2050 Szymon Hołownia
7. A different political grouping
8. None
9. Hard to say

M22. Have you been vaccinated against the flu in the 2020/2021 season?

1. Yes
2. No, I wanted to, but haven't been able to get vaccinated
3. No, because I don't want to get vaccinated
