# Supplementary material for: Trust in Medicine as a Factor Conditioning Behaviors Recommended by Healthcare Experts during the COVID-19 Pandemic in Poland
Source: Int J Environ Res Public Health. 2022 Jan 5;19(1):605. doi: 10.3390/ijerph19010605 (PMC8744838; doi:10.3390/ijerph19010605)
Supplement: Supplementary file 1 [file ijerph-19-00605-s001.zip › Supplementary Material B.pdf]

Appendix 2. Final cluster centers resulting from a cluster analysis using the k-means method (on a scale from 1 to 5)

	Focus	
	1	2
	**Moderate liberal (*N* = 471)**	**Moderate conservative (*N* = 601)**
	Centers of focus	
People who take part in the demonstrations are exposing society to an increase in coronavirus infections.	2	4
I think that demonstrations are necessary in order to make the government aware that many people do not agree with the Constitutional Tribunal's judgment	5	3
The Constitutional Tribunal's judgment was right	1	3
Polish abortion laws should be liberalized. Abortion should be available on demand.	4	3
It was not good timing for the Constitutional Tribunal to issue its judgment during the pandemic.	5	4
Poland's abortion laws should be maintained in their current state. The so-called abortion compromise should not be changed.	3	3
Protesters should not use profanities.	3	4
Protesters should not destroy church property.	4	4
Protesters should not offend priests and bishops.	2	4
